# Allicin shows antifungal efficacy against *Cryptococcus neoformans* by blocking the fungal cell membrane

**DOI:** 10.3389/fmicb.2022.1012516

**Published:** 2022-11-16

**Authors:** Zhun Li, Zhengtu Li, Jun Yang, Chun Lu, Yongming Li, Yinzhu Luo, Feng Cong, Rongmei Shi, Zhen Wang, Huaying Chen, Xinxia Li, Jinglu Yang, Feng Ye

**Affiliations:** ^1^State Key Laboratory of Respiratory Disease, The First Affiliated Hospital of Guangzhou Medical University, Guangzhou, China; ^2^Department of Respiratory and Critical Care Medicine, The Second Affiliated Hospital of Guangzhou Medical University, Guangzhou, China; ^3^College of Pharmacy, Xinjiang Medical University, Urumqi, China; ^4^Key Laboratory of Garlic Medical Research, Urumqi, China; ^5^School of Mechanical Engineering and Automation, Harbin Institute of Technology, Shenzhen, China

**Keywords:** allicin, antifungal, *Cryptococcus neoformans*, *Cryptococcosis*, cell membrane, activity, mechanism

## Abstract

Allicin, which is generated by the catalytic reaction between alliin and alliinase extracted from garlic, has been shown to have a wide range of antimicrobial activities, but its anti-*Cryptococcus* efficacy and mechanism are not quite clear. Here, we have determined that the Conversion rate of allicin in the reaction product reached 97.5%. The minimal inhibitory concentration (MIC) of allicin against *Cryptococcus neoformans (C. neoformans)* H99 was 2 μg/ml, which is comparable to fluconazole (FLU, 1 μg/ml). Furthermore, allicin exhibited effective antifungal activity against 46 clinical isolates of *C. neoformans*, and the MICs ranged from 1 to 8 μg/ml, even for AmB-insensitive strains. Interestingly, allicin also exerted additive or synergistic effects when combined with amphotericin B (AmB) and FLU. Time-killing curves and long-term live cell imaging of H99 showed that 4 MIC of allicin had fungicide activity. Additionally, allicin (4 and 8 mg/kg) exerted a dose-dependent therapeutic effect on H99-infected mice by significantly reducing the wet pulmonary coefficient and *Cryptococcus* load and reducing lung damage. Even the efficacy of 8 mg/kg was comparable to FLU (20 mg/kg). Transcriptomics revealed that allicin may act on the cell membrane of H99. Subsequently, transmission electron microscopy (TEM) observations showed that allicin clearly breached the cell membrane and organelles of H99. Confocal laser scanning microscopy (CLSM) results further confirmed that allicin disrupted the permeability of the cell membranes of H99 in a dose-dependent manner. Allicin exhibits strong anti-*C. neoformans* activity *in vitro* and *in vivo*, mainly by destroying the permeability and related functions of *Cryptococcus* cell membranes.

## Background

Garlic (*Allium sativum* L.; Family: Amaryllidaceae) is a well-known spice widely utilized for its medicinal properties and has been described in a wide range of ancient texts due to its widespread use throughout history for the treatment of various ailments and diseases (Choo et al., 2020). The main compound possessing antimicrobial activities in garlic, named allicin (S-(2-propenyl)-2-propene-1-sulfinothioate), is generated by the catalytic reaction between alliin and alliinase when garlic is crushed (Ankri and Mirelman, 1999; Nadeem et al., 2021). Allicin is a lipid-soluble sulfur compound (El-Saber Batiha et al., 2020), has a molecular formula of C_6_H_10_OS_2_ and a molecular weight of 162.27, can easily cross the cell membrane to fight microorganisms and has a broad antimicrobial spectrum based on its lipid solubility (Omar and Al-Wabel, 2010).

Allicin also reportedly exerts a wide range of properties, such as antiplatelet, antithrombosis, antioxidant, anti-inflammatory, immunomodulatory and neuroprotective activities (Shang et al., 2019; Asgharpour et al., 2021), and one of its most notable features is antimicrobial activity (Hunter et al., 2005). Furthermore, garlic extracts, including allicin, exert a broad spectrum fungicidal effect against a wide range of fungi, including *Candida*, *Torulopsis*, *Trichophyton*, *Cryptococcus*, *Aspergillus*, *Trichosporon*, *Rhodotorula* species and *Fusarium oxysporum* (Ogita et al., 2006; Parvu et al., 2019; El-Saber Batiha et al., 2020). Moreover, allicin and garlic oil show potent antifungal effects against *Candida albicans*, *Ascosphaera apis*, and *A. niger* (Kuda et al., 2004) and act by penetrating the cellular membrane as well as organelle membranes, such as the mitochondria, leading to organelle destruction and cell death (Pai and Platt, 1995). However, the efficacy and mechanism of allicin against *Cryptococcus* have not been defined.

*Cryptococcus* is widely distributed in nature, and isolation from pigeon dung is considered its main source of human infection (Franzot et al., 1999; Samarasinghe and Xu, 2018). *Cryptococcosis* resulting from *Cryptococcus* infection is one of the top three fungal infectious diseases in the world, approximately 1 million cases of C*ryptococcosis* are reported globally every year, especially common in Africa and the warm and humid tropical and subtropical southeast regions. And almost 625,000 people die from the disease, with a mortality rate of 20–70% (Park et al., 2009; Li et al., 2021). Moreover, *Cryptococcosis* patients undergo a long treatment cycle with a high cost, and AmB and FLU are the first-line clinical anti-*Cryptococcus* drugs. However, the existing antifungal drugs still have many problems that need to be solved, including drug toxicity and resistance (Coelho and Casadevall, 2016; Perfect, 2017; Rajasingham et al., 2017; Benedict et al., 2019). Therefore, the development of new drugs with high efficiency, low toxicity and low price is an urgent task. Some studies have shown that sodium houttuyfonate (SH), berberine (BER), jatrorrhizine (JAT), cinnamaldehyde (CIN) and other common active ingredients of traditional Chinese medicine have antifungal activity against *Candida albicans* and *Cryptococcus* (Arif et al., 2009; Di Santo, 2010). Therefore, the selection of new antifungal drugs from natural products will become a new strategy.

In conclusion, we expect to supplement the activity of allicin against *C. neoforman*s *in vivo* and *in vitro* and identify the mechanism *via* TEM and CLSM analyses. These results will provide a reference for the development of new antifungal drugs.

## Materials and methods

### Allicin reaction process and concentration analyses

Allicin was generated by the catalytic reaction between alliin (Xinjiang, batch no. AL160520, stored at 4°C) and alliinase (Xinjiang, batch no. 201801001, stored at −20°C) in this experiment. Allicin was mixed and stored at 4°C after ultrafiltration. Its purity was determined by high-performance liquid chromatography (HPLC, Shimadzu Corp, Japan). Twenty milligrams of hydroxybutyric acid was dissolved in 50% methanol and diluted with 100 ml of internal standard solution. Then, 290 mg of alliin and 580 mg of alliinase were mixed at a mass ratio of 1:2 with 10 ml of distilled water and reacted at room temperature for 30 min. Subsequently, 0.5 ml of the reaction solution was placed in a 25-ml flask, and 15 ml of methanol and 1.0 ml of internal standard solution were added. The mixtures were then diluted with 25 ml of a 1% formic acid solution, shaken well and filtered. A 20-μl aliquot of the solution was pipetted into a high-performance liquid chromatograph, a measurement was taken at UV 254 nm, and the amount of allicin produced was calculated.

### *Cryptococcus neoformans* strains and positive drugs

*Cryptococcus* standard strains (*C. neoforman*s H99), quality control strains (*Candida albicans* ATCC 22019 and *Candida albicans* ATCC 6258) and 46 clinical strains were obtained from the First Affiliated Hospital of Guangzhou Medical University. All clinical strains were identified as *C. neoforman*s by ink staining and MALDI-TOF mass spectrometry. All strains were incubated in Sabouraud dextrose agar (SDA, GuDuo, China) plates twice to ensure activity. After incubation at 35°C for 72 h, the concentration was prepared at (1–5) × 10^6^ cfu/ml, adjusted the inoculum to a McFarland standard of 0.5. The cells were diluted 50 times with RPMI-1640 culture and then 20 times (the final dilution was 1,000 times) to obtain a concentration between 1 and 5 × 10^3^ CFU/ml. AmB and FLU were dissolved in DMSO to prepare 1.6 mg/ml stock solutions and stored at −80°C until use.

### Minimal inhibitory concentration

The MICs were determined according to CLSI document M27 (Arastehfar et al., 2021). And the MIC was defined as the lowest concentration that substantially inhibited the fungi growth in the medium. MIC_50_ and MIC_90_ were defined as the MICs needed to inhibit 50 and 90% of fungi in a batch of experiments. The final concentrations of allicin were 0.0625–32 μg/ml, those of AmB were 0.03–16 μg/ml, and the concentrations of FLU were 0.125–64 μg/ml.A 0.5 McFarland turbidity standard suspension, corresponding to (1–5) × 10^3^ CFU/ml of each isolate was prepared. And incubated at 35°C for 72 h to observe the results. To ensure the accuracy of the experimental results, *Candida parapsilosis* ATCC 22019 and *Candida krusei* ATCC 6258 were included as quality control strains in the same test, and the results were interpreted according to CLSI document M60 (Tóth et al., 2019). Each experiment repeated three re-wells. When the results were inconsistent or the quality control strain was out of range, the experiment would be repeated again until the results of the three re-wells were consistent and at the same time in the quality control.

### Time-kill studies

An assay was performed to evaluate the time-kill kinetics of allicin against *C. neoforman*s. Allicin was diluted to 0.5 MIC, MIC, 2 MIC, 4 MIC, and 8 MIC in RPMI-1640 medium and cultured at 35°C with a starting inoculum concentration of 10^5^ cells/ml. A 100-μl aliquot was removed from every group and coated on SDA medium at 0, 2, 4, 6, 8, 10, and 12 h. The colony count during incubation at 35°C for various time points up to 72 h was calculated.

### Disk diffusion assay

To evaluate the combined efficacy of allicin with AmB or FLU, the fractional inhibitory concentration index (FICI) was calculated as FIC_A_ + FIC_B_, where FIC_A_ = MIC_drug A in combination_/MIC_drug A alone_ and FIC_B_ = MIC_drug B in combination_/MIC_drug B alone_ (Zhu et al., 2021). FICI ≤ 0.5 indicated that a synergistic interaction, FICI values of 0.5–1.0 indicated an additive interaction, FICI values of 1.0–2.0 indicated an irrelevant interaction, and FICI > 2.0 indicated an antagonistic interaction.

### *Cryptococcus* with live-cell imaging system

Long-term and continuous observations of living cells can be achieved with a live-cell imaging system. Based on the live-cell imaging system (Molecular Devices, San Jose, CA, United States), we first used an all-electric microscope (Olympus IX83, Tokyo, Japan) and Prime 95B SCMOS camera to obtain intermittent and continuous photos of *C. neoformans* and thus observe the changes in quantity and the short-term growth and division of *C. neoforman*s in the presence of allicin. The prepared cryptococcal solution was added to a 384-well plate (Thermo Scientific^™^ Nunc^™^; Sun et al., 2016), and solution with or without allicin was then added. The final concentration of *Cryptococcus* was 5 × 10^4^ cfu/ml, and the concentrations of allicin were 1 MIC, 2 MIC, and 4 MIC. The control group without increasing concentrations of allicin was observed at different time points with the live-cell imaging system. The division of each group of *C. neoforman*s was then calculated.

### Evaluation of the efficacy of allicin against pulmonary cryptococcosis in a mouse model

C57BL/6 female mice aged 6–8 weeks (animal license #: SCXK 2013–0002) were used for the *in vivo* experiments. *C. neoforman*s H99 that had grown for 3 days was inoculated with 5 × 10^6^ cfu/ml. The mice were anaesthetized with isoflurane and received 50 μl for nasal infection, and the control mice were anaesthetized with physiological saline. The study groups were administered fluconazole 20 mg/kg and allicin (4 and 8 mg/kg). All the animals were administered the drug treatment for 7 days after 4 h of infection.

After 7 days of treatment, the mice were humanely euthanized. The lungs were collected aseptically, washed, dried with filter paper and weighed. The wet pulmonary coefficient was calculated as follows: (lung weight/mouse body weight) × 100. The tissues were fully ground in 1 ml of PBS by magnetic beads. Homogenates were serially diluted 10-fold and then incubated on SDA plates at 35°C for 2 days to calculate the cfu values. An additional four mice were euthanized, and the lung tissues were isolated, fixed with a 4% formaldehyde solution for more than 24 h and then stained with hematoxylin-eosin (HE) and Grocott’s methenamine silver (GMS).

### RNA sequencing and bioinformatics analysis

A single *C. neoforman*s strain H99 was added to 10 ml of YPD liquid medium(ZeYe, China), incubated in constant shaker at 200 RPM and 37°C for 36 h, and then adjusted to 5 × 10^6^ cfu/ml. Allicin (2 MIC and 4 MIC) was added, and the untreated group was used as the blank control. The solutions were then shaken for 8 h and washed by centrifugation. The three groups of precipitates were frozen in liquid nitrogen and stored at −80°C.

*Cryptococcus* RNA was extracted with TRIzol (Life Technologies, United States). To ensure the purity of RNA, the OD value of nucleic acids was detected with a NanoDrop instrument, and samples with an A260/A280 ratio between 1.8 and 2.0 were used. The mRNA was reverse transcribed to obtain double-stranded cDNA, the double ends of cDNA were repaired, and the connector was then added for PCR amplification to construct the machine library. Prior to sequencing, library inspection was performed using a DNA 1000 Assay Kit (Agilent Technologies) to ensure the sequencing quality. Before bioinformation analysis, FASTP (Chen et al., 2018) was used for quality control to filter low-quality data. Clean reads were compared to the ribosome database of the species using the short read comparison tool Bowtie2 (Langmead and Salzberg, 2012) to remove part of the residual rRNA.

Gene expression pattern analysis was performed to cluster genes with similar expression patterns from multiple samples (at least 3 in a specific time point, space, or treatment dose size order). To examine the expression pattern of DEGs, the expression data of each sample (in the order of treatment) were normalized to 0, log2 (*v*1/*v*0), and log2(*v*2/*v*0) and then clustered by Short Time-series Expression Miner software (STEM; Ernst and Bar-Joseph, 2006). The parameters were set as follows: (1) the maximum unit change in model profiles between time points was 1; (2) the maximum output profile number was 20 (similar profiles were merged; and (3) the minimum ratio of fold change in DEGs was no less than 2.0. The clustered profiles with a *p* value ≤ 0.05 were considered significant profiles. The DEGs in all or each profile were then subjected to Gene Ontology (GO) and KEGG pathway enrichment analyses. Based on the hypothesis test with *p* value calculation and FDR correction, the GO terms or pathways with *Q* value ≤ 0.05 were defined as significantly enriched GO terms or pathways.

### Transmission electron microscopy

The ultrastructural changes in *C. neoforman*s strain H99 were observed through TEM (JEM-1400 PLUS, Tokyo, Japan). *C. neoforman*s suspensions and drugs were grouped as stated above. *C. neoforman*s were incubated in a shaker at 200 rpm and 37°C for 8 h with 2 MIC and 4 MIC allicin. The samples were then collected, centrifuged at 4,500 rcf for 5 min, washed once, slowly filled with 2.5% glutaraldehyde and fixed for more than 2 h. Dehydration, embedding, curing, slicing and staining were then performed. The samples were then analyzed by TEM.

### Confocal laser scanning microscopy

To further verify whether allicin damages the cell membrane, a LIVE/DEAD FungaLight^™^ Yeast Activity kit [Invitrogen, United States; containing two dye probes, propidium iodide (PI) and SYto-9], was used for validation (Luo et al., 2018). Eight hours after the *C. neoformans* strain H99 was exposed to 0.5 MIC and 1 MIC allicin in a 24-well plate, the cells were harvested and washed once with 1 ml of PBS. Then, 1 μl of PI and 1 μl of SYto-9 were added. The samples were incubated at 37°C for 20 min without light and fixed on slides for CLSM (Leica, Mannheim, Germany), and 488-nm and 544-nm lasers were used for imaging. The fluorescence intensity was analyzed using ImageJ software.

### Statistics

GraphPad Prism 8.0 and SPSS 18.0 software were used for data and image processing. The division of *C. neoforman*s was calculated by *T* test and one-way ANOVA. The colony counts, body weights and lung indices of mice were tested by ANOVA and Dunnett’s multiple comparison test. *p* < 0.05 was considered to indicate statistical significance. GO and KEGG analyses were performed using Fisher’s exact test, and only the categories and approaches with *p* < 0.05 were considered statistically significant.

## Results

### Analyses of the allicin contents

**Figure 1:**
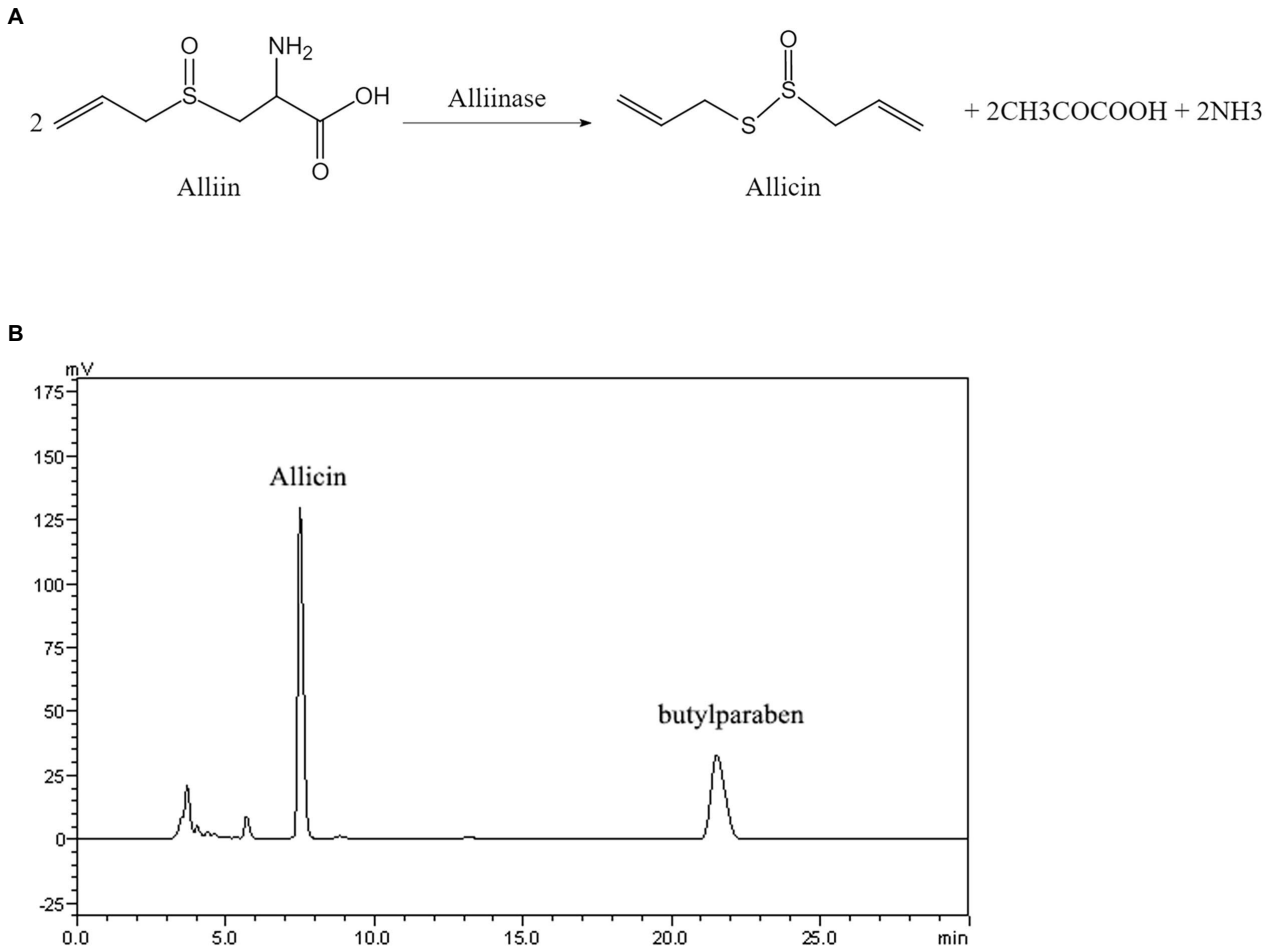
**(A)** Structural formula and reaction process diagram of allicin. **(B)** High-performance liquid chromatography (HPLC) chromatogram of allicin in the reaction solution composed of alliin and alliinase.

The molecular formula and reaction process of allicin are shown in Figure 1A. As demonstrated by HPLC analyses, the conversion rate of allicin reached 97.5% (Figure 1B). According to the molecular weight of the reaction, the conversion coefficient of allicin to allicin is 0.458, and the actual concentration of allicin can thus be obtained (Supplementary Material 1).

### Allicin shows good anti*-Cryptococcus neoformans* efficacy *in vitro*

As shown in Table 1, the MIC of allicin against *C. neoforman*s H99 was 2 μg/ml, which was comparable to that of fluconazole (FLU, 1 μg/ml) but somewhat different from that of amphotericin B (AmB, 0.125 μg/ml). Interestingly, the prodrugs alliin and alliinase of allicin did not show anti-*C. neoforman*s efficacy with MICs >16 μg/ml. Furthermore, allicin also has good antifungal activity against 46 clinical isolates of *C. neoforman*s, and the range of MICs was 1–8 μg/ml, which was also comparable to that of FLU (0.125–8 μg/ml) but higher than that of AmB (0.0625–0.5 μg/ml). More interestingly, allicin also retained better activity against AmB-resistant strains with MICs of 1–2 μg/ml (Table 1). Detailed MIC results for each strain are provided in (Supplementary Material 2).

**Table 1:** Minimal inhibitory concentration (MIC)-based comparison of allicin with AmB and FLU.

Group	*C. neoformans* H99	Clinical strains (44)			> ECV (2)
	MIC	MICs	MIC_50_	MIC_90_	MICs
Alliin	> 16				
Alliinase	> 16				
Allicin	2	1–8	2	8	1–2
AmB	0.125	0.0625–0.5	0.125	0.25	1–2
FLU	1	0.125–8	2	4	

The MIC evaluation criteria were as follows: AmB (100% inhibition of growth), FLU (50% inhibition of growth), and allicin (100% inhibition of growth). The interpretation criteria are described in the “Materials and methods” section.

### Allicin presents positive combined efficacy with AmB and FLU

The effects of allicin combined with AmB and FLU against *C. neoforman*s H99 were determined by the checkerboard microdilution method. The results showed that allicin combined with AmB exerted synergistic effects (FICI = 0.375), and these effects were 8 and 4 times lower than those of allicin or AmB alone. Allicin also exerted additive effects when combined with FLU (FICI = 0.625) against *C. neoforman*s H99, and the MICs were reduced 8- and 4-fold, respectively (Table 2). Furthermore, we also randomly selected three clinical *C. neoforman*s strains and found that allicin combined with AmB exerted additive effects, but with FLU showed irrelevant effects (Supplementary Material 3). Considering that each clinical strain may have different results due to different sources and drug sensitivities.

**Table 2:** Combined efficacy of allicin with AmB and FLU.

Strain	MIC		FIC		FICI	Result	MIC		FIC		FICI	Result
	Allicin	AmB	Allicin	AmB			Allicin	FLU	Allicin	FLU		
*C. neoformans* H99	2	0.25	0.25	0.0625	0.375	Synergy	2	2	0.25	1	0.625	Addition

### Inhibitory and fungicidal characteristics of different concentrations of allicin against *Cryptococcus neoformans* H99

**Figure 2:**
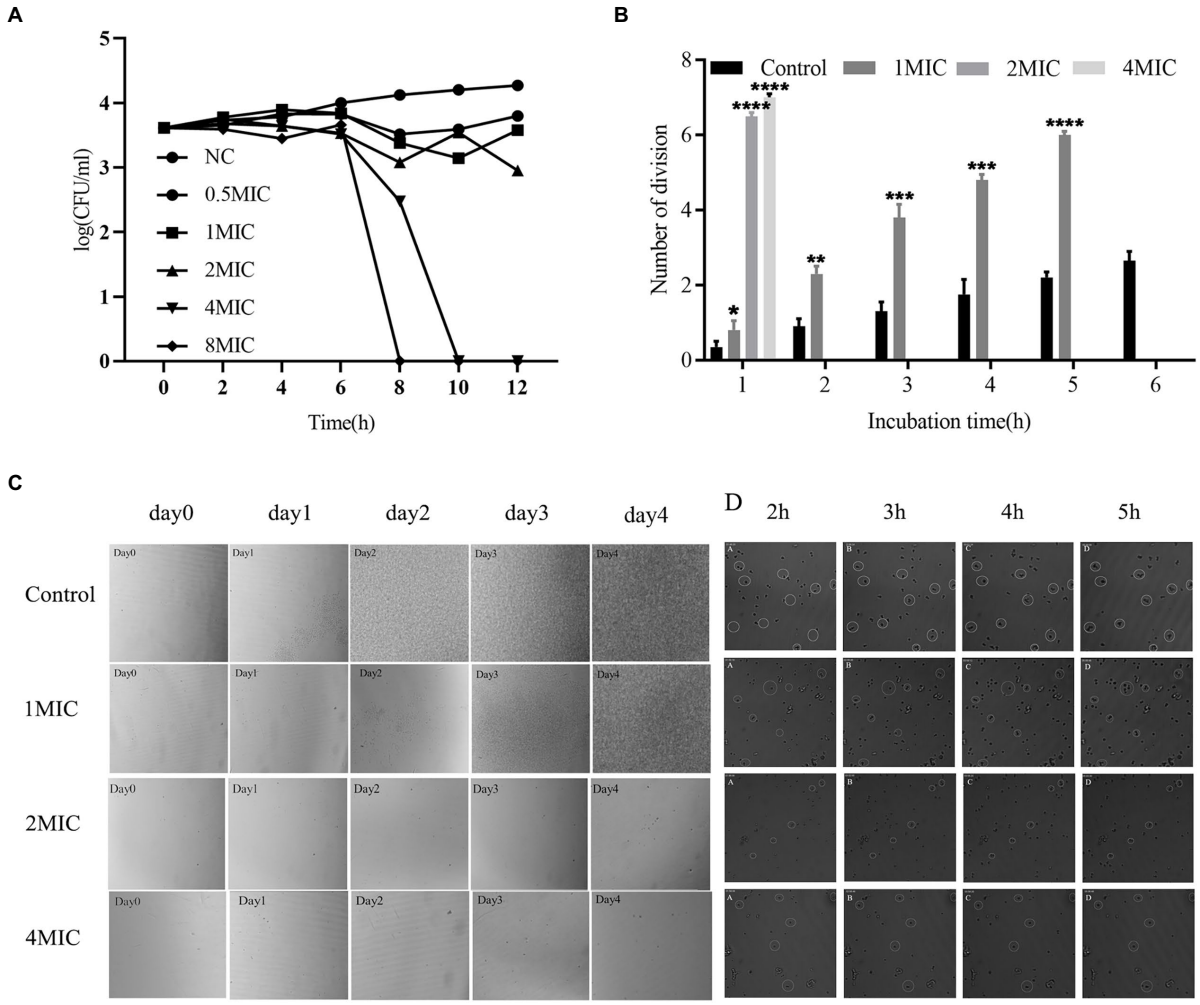
**(A)** Time-kill curve. The tested strain was *Cryptococcus neoformans* H99. The concentrations of allicin were NC (0), 0.5 MIC (1 μg/ml), 1 MIC (2 μg/ml), 2 MIC (4 μg/ml), 4 MIC (8 μg/ml) and 8 MIC (16 μg/ml). The colonies were counted after incubation for 48 h. **(B)** Growth time and division of *C. neoformans* H99. *Cryptococcus* continued to grow in the control group, allicin at 1 MIC significantly inhibited growth, allicin at 2 MIC and 4 MIC significantly prolonged the division of *Cryptococcus* within 1 h, and no division was observed after incubation for 2 h. **(C)** Real-time imaging of living *C. neoformans* H99. Continuous photographs were obtained to observe the overall growth of *C. neoformans* H99 after long-term culture in the presence of allicin (left). The cells were incubated for 24, 48, 72, and 96 h, and the long-term growth changes in *Cryptococcus* were observed. **(D)** The inhibitory effect of allicin on the proliferation and division of *Cryptococcus* (right) was observed by continuous photographs at 2, 3, 4, and 5 h.

The time-kill curve for 0–12 h demonstrated that allicin at 0.5 MIC, 1 MIC and 2 MIC exerted an inhibitory effect on *C. neoforman*s H99. However, 4 MIC and 8 MIC (8 and 16 μg/ml) exerted fungicidal effects (Figure 2A). The fungicidal effect of allicin was positively correlated with the concentration of allicin.

To further verify the efficacy characteristics of allicin against *C. neoforman*s, real-time imaging of living *C. neoforman*s H99 was performed. The control group showed that *C. neoforman*s was slightly increased at 24 h and significant increased at 48 h, 72 h and 96 h. After treatment with allicin at 1 MIC for 24 h, *C. neoforman*s division and growth were basically not observed, and the colonies had grown to cover the entire field of vision after 96 h. However, the growth of *C. neoforman*s was almost stagnant after the 2 MIC treatment, and no significant increase was observed after 96 h. In addition, the 4 MIC treatment yielded results similar to those found with the 2 MIC treatment (Figure 2C). The long-term culture results indicated that 1 MIC allicin may exert an inhibitory effect on *Cryptococcus*, whereas 2 MIC and 4 MIC allicin may have fungicidal effects.

To further observe the short-term effect of allicin on the proliferation and division of *C. neoforman*s, the cells were continuously photographed and observed for 6 h. The untreated group divided rapidly within 6 h and underwent a division almost every 40 min. The growth rate of *C. neoforman*s due to division was significantly reduced by the 1 MIC treatment, and a division occurred every 1.5 h. Two hours later, no obvious *C. neoforman*s division or growth was observed after treatment with 2 MIC or 4 MIC allicin (Figure 2B). The fission growth was completely inhibited with no division by allicin at 4 MIC, and large fragments of *C. neoforman*s were found (Figure 2D). The results were consistent with the discontinuous photographing during long-term incubation and with the results from the time-kill curve. In short, allicin at 4 MIC exerts fungicidal effects in a short time, whereas allicin at 2 MIC needs a long time to achieve this effect. A dynamic video is shown in Supplementary Material 4.

### Allicin also shows efficacy against *Cryptococcus neoformans* in a mouse model

**Figure 3:**
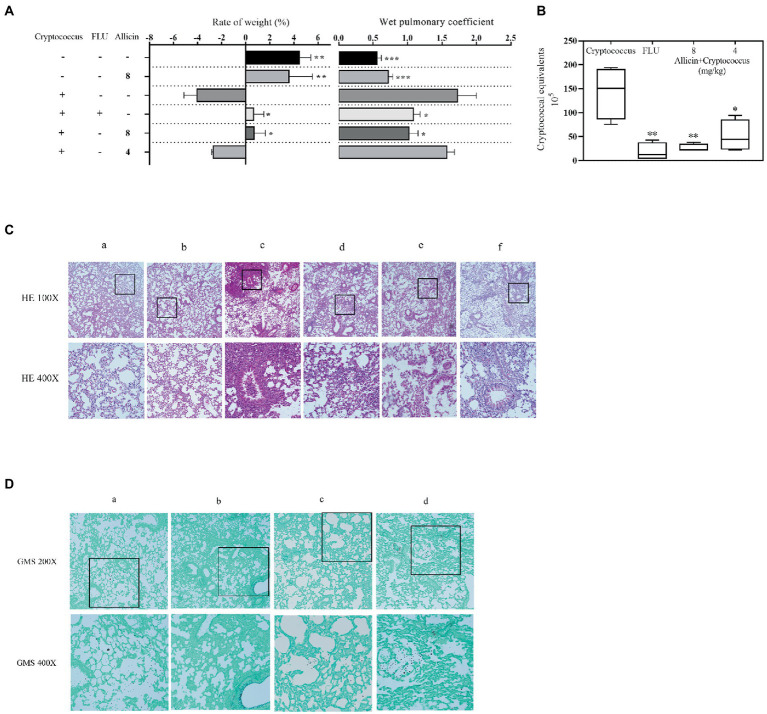
**(A)** Mice were treated with 50 μl of a 5 × 106 cfu/ml *C. neoformans* H99 suspension for intranasal infection and continuously administered drugs for 7 days. After 7 days of treatment, the weight change rate was calculated, and the weight change rate of the mice was calculated as follows: (weight on the seventh day – weight on the day of infection)/weight on the day of infection × 100. The weights of the lungs of all experimental mice (5 in each group) were weighed, and the wet pulmonary coefficient of each mouse was calculated. **(B)** The *Cryptococcus* burden in the lungs of mice treated with 20 mg/kg FLU, 4 mg/kg allicin or 8 mg/kg allicin was measured on the seventh day. CFUs with different dilutions were counted after 48 h of incubation. ANOVA and Dunnett’s multiple comparisons were used to analyze the differences between the results. (^*^p < 0.05; ^*^p < 0.01; ^***^p < 0.001). **(C)** Lung histopathological sections were stained with HE and GMS. For the HE staining, a shows the normal uninfected group (PBS), b shows the uninfected and 8 mg/kg allicin groups, c shows the model group, d shows the group treated with 20 mg/kg FLU, and e and f show the 8 and 4 mg/kg allicin groups, respectively. **(D)** For the GMS staining, a shows the model group, b shows the 20 mg/kg FLU group, and c and d show the 8 and 4 mg/kg allicin groups, respectively.

The results showed that the weight of the *C. pneumoniae* model group was significantly decreased compared with that of the normal group, the weight change rate was significantly reduced after treatment with FLU and 8 mg/kg allicin compared with that of the model group (*p* < 0.05), and the efficacy of FLU was similar to that of 8 mg/kg allicin. However, no significant reducing was observed in the 4 mg/kg allicin-treated groups compared with the model group (*p* > 0.05). Furthermore, no significant change in body weight was found in the group treated with the drug alone compared with the control group (Figure 3A). Similarly, the wet pulmonary coefficient of the model group was significantly higher than that of the normal group. The 8 mg/kg allicin treatment significantly reduced the wet pulmonary coefficient compared with that of the model group, and the resulting index was equivalent to that found after FLU treatment. Although the 4 mg/kg allicin treatment also reduced the wet pulmonary coefficient in mice, the difference was not significant (Figure 3A).

The *C. neoforman*s burden in the lungs was significantly lower in all drug treatment groups than in the model group, and no significant difference was found between the 4 and 8 mg/kg allicin groups and the FLU group (Figure 3B).

The lung pathological staining results showed that the pulmonary bronchial epithelial cells exhibited a normal morphology in the noninfected 8 mg/kg allicin group, and no significant difference was found between this group and the normal group. In the model group, many inflammatory cells infiltrated the alveolar cavity and trachea, the alveoli were destroyed (Figure 3C), and extensive infiltration of *C. neoforman*s was observed. However, the FLU and 8 mg/kg allicin groups showed significant reducing in lung lesions and a small amount of inflammatory cell infiltration (Figure 3C). In addition, *C. neoforman*s was significantly reduced and observed only in local lung tissues (Figure 3D). The 4 mg/kg allicin group also showed significantly reduced lung consolidation and *Cryptococcus* infiltration, but a small number of inflammatory cells and *C. neoforman*s were still observed in the tracheal cavity. As a result, increasing dosages of allicin reduced the pathology of the mouse model (Figures 3C,D).

### Transcriptomics reveals that allicin mainly affects the cell membrane function of *Cryptococcus neoformans*

**Figure 4:**
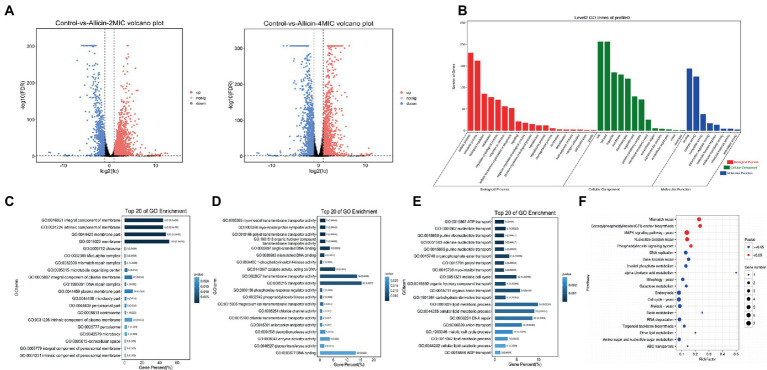
**(A)** Volcano plots of the genes that shows significantly differential expression after the 2 MIC and 4 MIC of alliin treatments compared with the normal group. The closer the genes are to the two ends, the greater the degree of difference. **(B)** GO map of putative target genes. The three colors describe the molecular function, cellular component and biological process of the genes. **(C,D,E)** GO enrichment bar graph. The first 20 GO terms with the smallest p value were used to draw the graph, the ordinate is the GO term, and the abscissa is the percentage of the number of GO terms for all differential genes. A darker color indicates a smaller *p* value, and the value on the column is the number of GO terms and p value. From left to right are the enrichment of cell components **(C)**, molecular functions **(D)** and biological processes **(E)**. **(F)** KO enrichment bubble graph. The first 20 pathways with the smallest *p* values were used to draw the map. The ordinate was the pathway, and the abscissa was the enrichment factor; that is, the differentially expressed genes in this pathway were divided by all the numbers. The size indicates the number, and a redder color indicates a smaller *p* value.

RNA sequencing revealed many differentially expressed genes in *C. neoforman*s H99 treated with 2 MIC and 4 MIC allicin, 3,061 and 2,774 differentially expressed genes, respectively, were detected. The differential gene expression patterns are illustrated in a volcano plot (Figure 4A). To conduct a cluster analysis of the gene expression curve of *C. neoforman*s in the presence of allicin at different concentrations, we selected the control group without allicin and the groups treated with allicin at 2 MIC and 4 MIC for trend analysis (Figure 4). The results showed that the expression of 417 genes in one trend pattern continued to decrease, and all genes with significant differences were found to be related to membranes in the GO functional enrichment analysis. The enriched molecular functions were mainly related to transmembrane transporter activity and DNA binding, and the biological process involved lipid metabolic processes and DNA repair (Figures 4B–E).

Subsequently, a KEGG pathway analysis of the identified genes was performed to further elucidate the most important biochemical metabolic pathways and signal transduction pathways involving the differentially expressed genes. We found that the main enrichment pathways were mismatch repair, glycosylphosphatidylinositol (GPI)-anchor biosynthesis, MAPK signaling pathway-yeast, nucleotide excision repair, and the phosphatidylinositol signaling system (Figure 4F). The top 50 genes that differed significantly between each group are listed in Supplementary Material 5.

### Allicin acts on the membrane of *Cryptococcus neoformans* by disrupting its permeability

**Figure 5:**
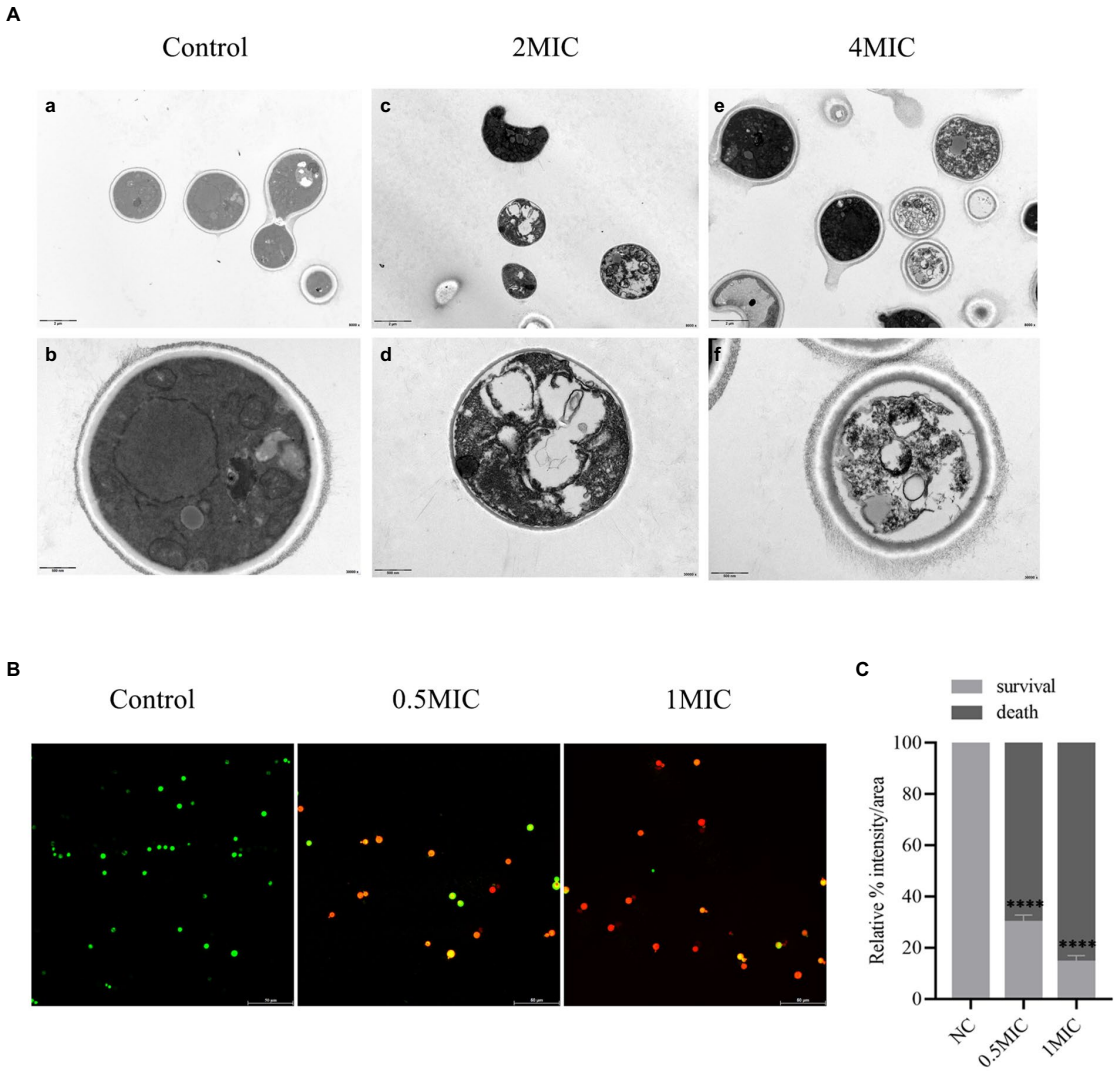
**(A)** TEM. (a,b) show the normal structures of *C. neoformans* H99. (c,d) show the 2 MIC allicin group; (e,f) show the 4 MIC allicin group. The internal structure of each group was observed by 800 × and 30,000 × electron microscopy. **(B)** Mixed dyeing was performed with PI and SYto-9 dyes. Yeasts with intact membranes show green fluorescence, whereas those with damaged membranes emit red fluorescence. **(C)** The fluorescence intensity of each CLSM image was analyzed using ImageJ software. *p* value: ^****^p < 0.0001.

To further verify whether allicin acted on the membrane of *Cryptococcus*, the structural change in *C. neoforman*s H99 was observed by TEM. After 8 h of treatment with allicin at 2 MIC, the *C. neoforman*s H99 capsule disappeared, cells and organelles were deformed, some cells even underwent cell wall rupture leading to cytoplasmic overflow, and a few organelles, including the nucleus and mitochondria, were dissolved and necrotic. However, the 4 MIC group showed significantly different results, and most of the *C. neoforman*s had complete pods. However, a large number of electron-dense bodies appeared in the cells, organelles were widely dissolved and necrotic, and some organelles even disappeared completely (Figure 5A).

Furthermore, the effect of allicin on the membrane permeability of *Cryptococcus* was detected by CLSM. The results showed that a large amount of green fluorescence was observed in the control group after cultivation for 8 h, whereas after treatment with allicin at 0.5 MIC, the amount of green fluorescence *C. neoforman*s was significantly decreased, and the amount of red fluorescence was significantly increased. Almost no green fluorescence was observed with the 1 MIC treatment (Figure 5B). This finding was also confirmed by the quantitative fluorescence intensity of each group, which revealed that allicin intervention significantly reduced the number of surviving *C. neoforman*s (Figure 5C). This finding suggests that damage to the membrane of *C. neoforman*s is stronger with increasing doses of allicin.

## Discussion

The purpose of this study was to explore the activity and mechanism of allicin, which is generated by the catalytic reaction between alliin and alliinase, against *Cryptococcus*. The study confirms that allicin exhibited effective concentration-dependent anti-*C. neoforman*s activity *in vitro* and *in vivo*. Furthermore, the anti-*C. neoforman*s effect of a common dose (8 mg/kg) was similar to that of FLU (20 mg/kg) in a mouse model. Interestingly, the alliin and alliinase prodrugs did not show anti-*C. neoforman*s efficacy *in vitro*. Moreover, the mechanism of allicin against *C. neoforman*s may affect the expression of cell membrane-related pathways by disrupting the permeability of the *C. neoforman*s cell membrane and thus affecting the normal function of the cell membrane.

Allicin is a new natural compound that is isolated and purified from the natural plant garlic (Borlinghaus et al., 2014). The results of our study also indicate that allicin has strong antifungal activity against *C. neoforman*s *in vitro* and *in vivo* (Table 1; Figure 3), even though no criteria for determining the efficacy of allicin against *C. neoforman*s are provided in CLSI document M27-A3. In addition, allicin at a common dose of 8 mg/kg has anti-*C. neoforman*s activity *in vitro* and *in vivo* similar to that of FLU (20 mg/kg) in a mouse pulmonary *Cryptococcosis* model (Figure 3). Previous research also showed that the MIC of allicin against *A. fumigatus* ATCC 36607 is 3.2 μg/ml, similar to our result (Choo et al., 2020). Moreover, the MICs of the positive controls FLU and AmB were also in accordance with the results of a multicentre study performed in China (Fan et al., 2016). Additionally, other studies have shown that other components of garlic also have antifungal activity (El-Saber Batiha et al., 2020), including activity against *Cryptococcus*. It is worth noting that the allicin used in this study was generated by the catalytic reaction between alliin and alliinase, and the conversion rate of allicin reached 97.5% (Figure 1). Furthermore, our findings confirmed that the prodrugs alliin and alliinase did not show anti-*C. neoforman*s efficacy *in vitro* (Table 1). Therefore, these results clearly confirm that allicin has strong antifungal activity against *C. neoforman*s.

Additionally, the results of this study showed that allicin exhibits positive combined efficacy with AmB against *C. neoforman*s (Table 2). But with FLU, the combination was not quite the same during *C. neoforman*s H99 and other Clinical strains. The final results of drug sensitivity may be different because of the different sources and virulence stability of different clinical strains. Of course, this needs to be verified by a large number of clinical strains. But overall, this finding may reveal one of the clinical applications of allicin against *C. neoformans*. Other studies have also shown that allicin or other active constituents of garlic have good antimicrobial properties when used in combination with other medications (Choo et al., 2020). Combination drugs may not only reduce the amount of each individual drug but also be beneficial in the treatment of *Cryptococcus* infected by FLU- or AmB-resistant strains (Meletiadis et al., 2005). Of course, combination antifungal therapy could reduce antifungal killing and clinical efficacy, increase the potential of drug interactions and drug toxicities, and carry a much higher cost for antifungal drug expenditures without a proven clinical benefit (Lewis and Kontoyiannis, 2001). Thus, more studies are needed to confirm whether allicin and other antifungal drugs have good combined efficacy.

Our study found that allicin can affect the expression of cell membrane-related pathways by disrupting the permeability of the *C. neoforman*s cell membrane while affecting the normal function of the cell membrane (Figures 4, 5). Other studies have also shown that allicin and garlic oil exhibits potent antifungal effects by penetrating the cellular membrane as well as organelle membranes, such as the mitochondria, leading to organelle destruction and cell death (Pai and Platt, 1995). In recent years, the role of the fungal cell membrane in pathogenesis and the identification of targets to improve therapeutic methods have been the focus of research (Muller et al., 2013), and significant progress has been made. For example, triazole antifungal agents, including itraconazole (Kurn and Wadhwa, 2021), voriconazole (Lee et al., 2019) and posaconazole (Howard et al., 2011), which act on the target of the fungal cell membrane, change the fluidity and permeability of the cell membrane and eventually cause the fungus to die. Additionally, a transcriptomic analysis found that the target genes of allicin treatment focus on mismatch repair, GPI-anchor biosynthesis, MAPK signaling pathway-yeast and the phosphatidylinositol signaling system (Figure 4), which are associated with the fungal cell membrane. GPI-anchored proteins play an important role in fungal adhesion, morphological transformation and cell wall synthesis (Djordjevic, 2010) and have great potential as targets in anti-*Cryptococcus* therapy. Jawsamycin, also from a natural product, was found to have antifungal properties by inhibiting the biosynthesis of GPI-anchored proteins (Fu et al., 2020). Moreover, the MAPK pathway is involved in the regulation of morphological and structural integrity and melanin formation in *Cryptococcus* and is critical for the pathogenesis of *Cryptococcus* (Alspaugh et al., 2000). In addition, Fludioxonil can also achieve antifungal effects by activating the MAPK signaling pathway and causing morphological defects in *Cryptococcus* (Kojima et al., 2006). Our results also found that allicin can destroy the cell membrane permeability of *C. neoforman*s and we have found some possible pathways through transcriptome, combined with existing studies, we believe that these pathways are likely to become future targets of *Cryptococcus.* The activity and mechanism of allicin were preliminarily explored in this study. However, this study has some disadvantages. The main shortcoming is that the specific signaling pathway and target of allicin are to be verified but it will be the focus of our follow-up research work.

## Conclusion

In this study, we found that allicin has effective anti-*C. neoforman*s activity *in vitro* and *in vivo* and can be used in combination with traditional anti-*Cryptococcus* chemical drugs to reduce the drug dosages. The mechanism of allicin is disruption of the membrane permeability of *Cryptococcus* and affecting the pathways related to mismatch repair and GPI-anchor biosynthesis, as well as the MAPK signaling pathway in yeast and the phosphatidylinositol signaling system in the membrane, which results in abnormal function and anti-*Cryptococcus* activity. These pathways are the direction that we would like to further study. And in the future, more in-depth research can be conducted on the effect of allicin combined with other drugs. The cell membrane is a hot spot in the development of new antifungal drugs, and further exploration of its specific targets is worthwhile. This study provides a reference for the subsequent development of novel anti-cryptococcal drugs and for the clinical formulation of a new combination of drugs.

## Data availability statement

The raw sequence data reported in this paper have been deposited in the Genome Sequence Archive (Genomics, Proteomics & Bioinformatics 2021) in National Genomics Data Center (Nucleic Acids Res 2022), China National Center for Bioinformation / Beijing Institute of Genomics, Chinese Academy of Sciences (GSA-Human: HRA003391) that are publicly accessible at https://ngdc.cncb.ac.cn/gsa-human.

## Ethics statement

The animal study was reviewed and approved by the Care and Use Committee of Guangdong Laboratory Animals Monitoring Institute (Approval ID SCXK 2013-0002).

## Author contributions

ZuL, ZeL, and FY conceived the study and designed the experiment. ZuL, CL, YML, YZL, FC, ZW, and HC performed the experiments. ZuL, ZeL, and JY analysed the data and drafted the manuscript. All authors fulfilled the contribution requirements as per the International Committee of Medical Journal Editors role of authors and contributor guidelines. All authors contributed to the article and approved the submitted version.

## Funding

This work was funded by the independent fund of State Key Laboratory of Respiratory Diseases (SKLRD-Z-202019) and The Guangzhou Institute of Respiratory Health Open Project (2019GIRHZ06), and partial funding was obtained from Xinjiang Elexin Pharmaceutical Co. LTD.

## Conflict of interest

The authors declare that the research was conducted in the absence of any commercial or financial relationships that could be construed as a potential conflict of interest.

## Publisher’s note

All claims expressed in this article are solely those of the authors and do not necessarily represent those of their affiliated organizations, or those of the publisher, the editors and the reviewers. Any product that may be evaluated in this article, or claim that may be made by its manufacturer, is not guaranteed or endorsed by the publisher.

## Supplementary material

The Supplementary material for this article can be found online at: https://www.frontiersin.org/articles/10.3389/fmicb.2022.1012516/full#supplementary-material

SUPPLEMENTARY FIGURE S1 Results of the allicin content determinations. AX is the peak area of themain peak of the test solution, and AR is the peak area of the main peak of the alanine control solution.

## Abbreviations

AmB: AmphotericinB
FLU: Fluconazole
SH: Sodiumhouttuyfonate
BER: Berberine
JAT: Jatrorrhizine
CIN: Cinnamaldehyde
TEM: Transmission electron microscopy
CLSM: Confocal laser scanning microscopy
MIC: Minimal inhibitory concentration
FICI: fractional inhibitory concentration index
SDA: Sabouraud dextrose agar
HE: hematoxylin-eosin
GMS: Grocott’s methenamine silver
GO: Gene Ontology
STEM: Short Time-series Expression Miner software
