# Supplementary figures and images for: Allicin shows antifungal efficacy against Cryptococcus neoformans by blocking the fungal cell membrane
Source: Front Microbiol. 2022 Nov 16;13:1012516. doi: 10.3389/fmicb.2022.1012516 (PMC9709445; doi:10.3389/fmicb.2022.1012516)

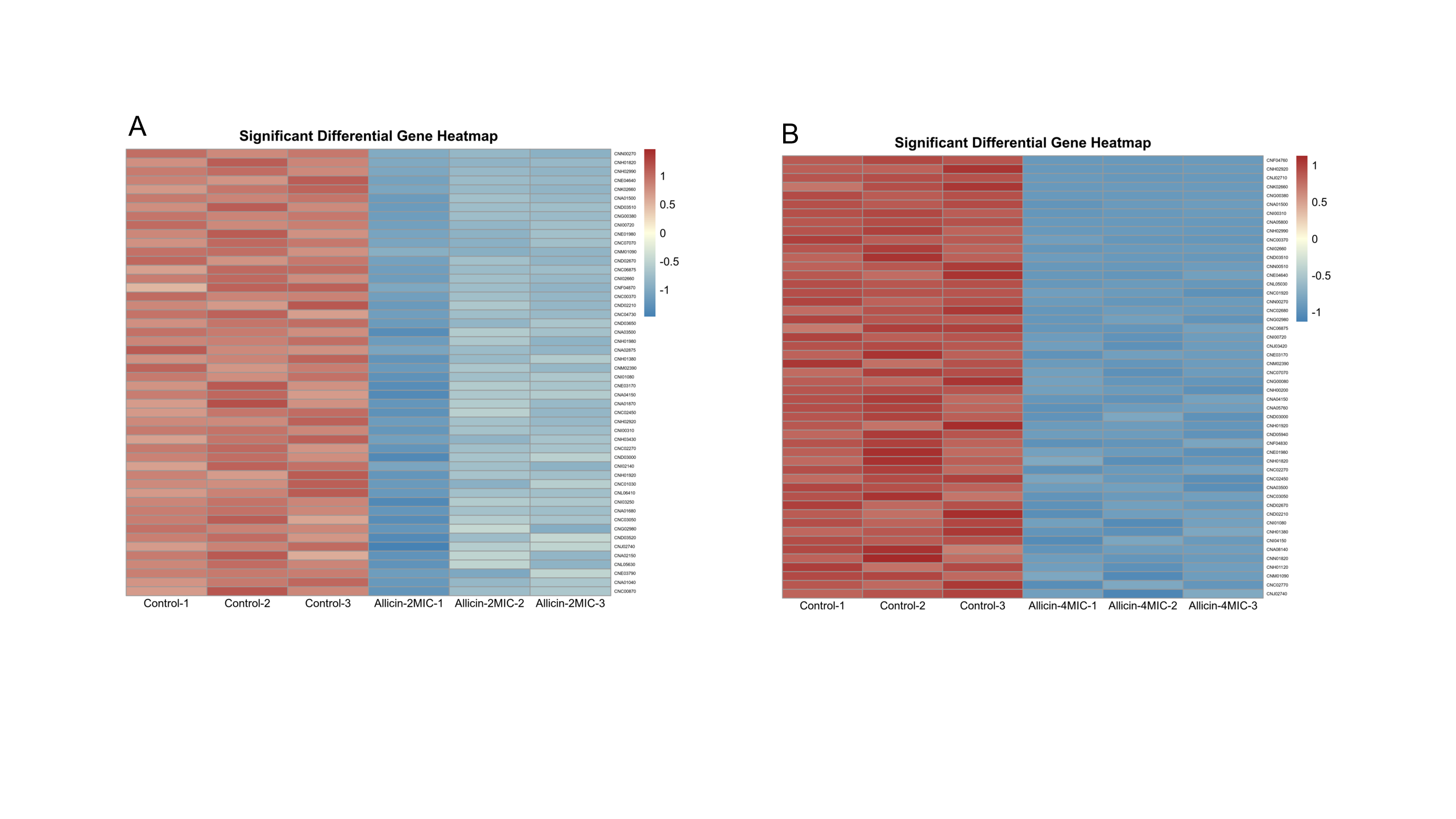

Supplement: SUPPLEMENTARY FIGURE S1 — Results of the allicin content determinations. AX is the peak area of themain peak of the test solution, and AR is the peak area of the main peak of the alanine control solution. [file Image_1.TIF]
