# Supplementary material for: Allicin shows antifungal efficacy against Cryptococcus neoformans by blocking the fungal cell membrane
Source: Front Microbiol. 2022 Nov 16;13:1012516. doi: 10.3389/fmicb.2022.1012516 (PMC9709445; doi:10.3389/fmicb.2022.1012516)
Supplement: Supplementary file 3 [file Table_2.DOCX]

**Figure S2**. Details of MIC comparison of allicin with AmB and FLU are as follows.MICs that less than or equal to the ECV were defined as wild-type (WT) of *C.neoformans.* and greater than ECV as non-WT strains.

| *C.neoformans* | MIC(μg/ml） | | | | |
| --- | --- | --- | --- | --- | --- |
|  | Allicin | AmB | results | FLU | results |
| 1 | 1 | 0.125 | <ECV | 2 | <ECV |
| 2 | 1 | 0.125 | <ECV | 4 | <ECV |
| 3 | 1 | 0.125 | <ECV | 4 | <ECV |
| 4 | 1 | 0.125 | <ECV | 4 | <ECV |
| 5 | 1 | 0.125 | <ECV | 2 | <ECV |
| 6 | 1 | 0.125 | <ECV | 4 | <ECV |
| 7 | 1 | 0.125 | <ECV | 4 | <ECV |
| 8 | 2 | 0.25 | <ECV | 4 | <ECV |
| 9 | 1 | 0.125 | <ECV | 4 | <ECV |
| 10 | 1 | 0.25 | <ECV | 4 | <ECV |
| 11 | 1 | 0.125 | <ECV | 4 | <ECV |
| 12 | 1 | 0.25 | <ECV | 4 | <ECV |
| 13 | 2 | 0.25 | <ECV | 4 | <ECV |
| 14 | 2 | 0.25 | <ECV | 4 | <ECV |
| 15 | 1 | 0.125 | <ECV | 0.5 | <ECV |
| 16 | 2 | 0.125 | <ECV | 4 | <ECV |
| 17 | 2 | 0.25 | <ECV | 4 | <ECV |
| 18 | 1 | 1 | >ECV | 0.25 | <ECV |
| 19 | 2 | 0.5 | ECV | 0.125 | <ECV |
| 20 | 2 | 2 | >ECV | 4 | <ECV |
| 21 | 1 | 0.25 | <ECV | 2 | <ECV |
| 22 | 2 | 0.125 | <ECV | 4 | <ECV |
| 23 | 2 | 0.25 | <ECV | 1 | <ECV |
| 24 | 2 | 0.125 | <ECV | 1 | <ECV |
| 25 | 8 | 0.125 | <ECV | 2 | <ECV |
| 26 | 8 | 0.125 | <ECV | 2 | <ECV |
| 27 | 8 | 0.25 | <ECV | 2 | <ECV |
| 28 | 8 | 0.0625 | <ECV | 2 | <ECV |
| 29 | 8 | 0.0625 | <ECV | 2 | <ECV |
| 30 | 8 | 0.0625 | <ECV | 2 | <ECV |
| 31 | 8 | 0.25 | <ECV | 2 | <ECV |
| 32 | 8 | 0.25 | <ECV | 4 | <ECV |
| 33 | 8 | 0.25 | <ECV | 2 | <ECV |
| 34 | 8 | 0.25 | <ECV | 1 | <ECV |
| 35 | 4 | 0.0625 | <ECV | 1 | <ECV |
| 36 | 4 | 0.0625 | <ECV | 0.5 | <ECV |
| 37 | 8 | 0.125 | <ECV | 1 | <ECV |
| 38 | 8 | 0.25 | <ECV | 2 | <ECV |
| 39 | 8 | 0.125 | <ECV | 2 | <ECV |
| 40 | 8 | 0.125 | <ECV | 8 | ECV |
| 41 | 8 | 0.125 | <ECV | 4 | <ECV |
| 42 | 8 | 0.5 | ECV | 1 | <ECV |
| 43 | 8 | 0.25 | <ECV | 2 | <ECV |
| 44 | 8 | 0.125 | <ECV | 2 | <ECV |
| 45 | 2 | 0.25 | <ECV | 1 | <ECV |
| 46 | 2 | 0.5 | ECV | 2 | <ECV |
| *C.neoformans* H99 | 2 | 0.125 | <ECV | 1 | <ECV |
