# Supplementary material for: Allicin shows antifungal efficacy against Cryptococcus neoformans by blocking the fungal cell membrane
Source: Front Microbiol. 2022 Nov 16;13:1012516. doi: 10.3389/fmicb.2022.1012516 (PMC9709445; doi:10.3389/fmicb.2022.1012516)
Supplement: Supplementary file 4 [file Table_3.DOCX]

**Table S3(A).** Combined efficacy of allicin with AmB against clinical strains.

| Strains | MIC | | Combination | | FICI | Results |
| --- | --- | --- | --- | --- | --- | --- |
|  | Allicin | AMB | Allicin | AMB |  |  |
| *C.neoformans* | 2 | 0.5 | 1 | 0.125 | 0.75 | additive |
| *C.neoformans* | 1 | 0.125 | 0.5 | 0.0625 | 1 | additive |
| *C.neoformans* | 8 | 0.25 | 2 | 0.125 | 0.75 | additive |

**Table S3(B).** Combined efficacy of allicin with FLU against clinical strains.

| Strains | MIC | | Combination | | FICI | Results |
| --- | --- | --- | --- | --- | --- | --- |
|  | Allicin | FLU | Allicin | FLU |  |  |
| *C.neoformans* | 2 | 2 | 2 | 2 | 2 | irrelevant |
| *C.neoformans* | 1 | 4 | 1 | 4 | 2 | irrelevant |
| *C.neoformans* | 8 | 2 | 8 | 2 | 2 | irrelevant |
